# Sakuranetin Reveals Potent, Stage-Dependent Anthelmintic Activity against *Angiostrongylus cantonensis*

**DOI:** 10.1021/acsomega.6c04512

**Published:** 2026-07-15

**Authors:** Regina C. Lourenço, Lucas Fukui-Silva, Monique C. Amaro, Dalete Christine S. Souza, Pedro Enrico H. Tesser, Erica Fernanda da S. Tirelli, João Henrique G. Lago, Josué de Moraes

**Affiliations:** † Research Center on Neglected Diseases, 149945 Brazil University, São Paulo 08230-030, SP, Brazil; ‡ Research Center on Neglected Diseases, 92928 Guarulhos University, Guarulhos 07023-070, SP, Brazil; § Center for Natural and Human Sciences, Federal University of ABC, São Paulo 09210-180, SP, Brazil

## Abstract

*Angiostrongylus cantonensis*, the leading cause of human eosinophilic meningitis, is an emerging zoonotic parasite of growing public health concern. Current therapeutic options show limited efficacy, particularly against larval stages, underscoring the need for new anthelmintic agents. Natural products derived from Brazilian biodiversity represent a promising source for discovery of new bioactive compounds. In this study, we evaluated the anthelmintic activity of sakuranetin, a naturally occurring flavanone from Brazilian plant *Baccharis lateralis* (Asteraceae), against first-stage (L1) and third-stage (L3) larvae of *A. cantonensis*, along with its safety profile. Sakuranetin exhibited stage-dependent activity, being more active against L1 larvae (EC_50_ = 8.1 μM) than against L3 (EC_50_ = 62.7 μM), outperforming albendazole (∼15 μM) and showing comparable activity to pyrantel pamoate (∼10 μM) in L1. No cytotoxic effects were observed in mammalian cell lines up to 500 μM, resulting in high selectivity indices, and no toxicity was detected in *Caenorhabditis elegans*. Morphological and morphometric analyses revealed pronounced phenotypic alterations, including body contraction and significant reduction in larval length. Fluorescence microscopy revealed a stage-dependent increase in propidium iodide staining, with L1 larvae showing markedly higher fluorescence than L3, consistent with greater susceptibility and reduced viability. *In silico* analysis did not identify relevant alerts for hepatotoxicity or neurotoxicity. Collectively, these findings demonstrate that sakuranetin exhibits stage-dependent anthelmintic activity against *A. cantonensis*, with low toxicity in the experimental models evaluated and favorable predictive toxicological properties, supporting its potential as a candidate for antiparasitic drug development.

## Graphical abstract

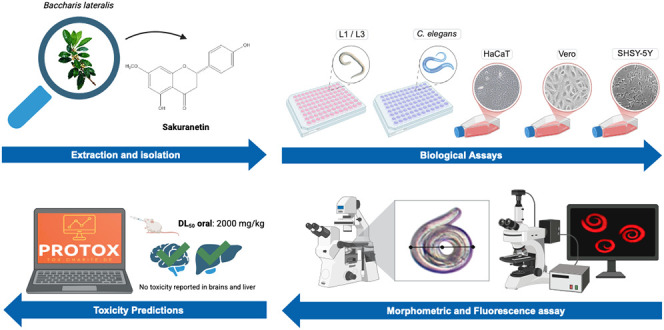

## 1 Introduction

Parasitic nematode infections represent a major global public health burden, affecting millions of people worldwide.^1,2^ Among these, *Angiostrongylus cantonensis* (rat lungworm) has emerged as a zoonotic parasite of increasing clinical relevance.^3^ The life cycle of *A. cantonensis* involves rodents as definitive hosts and terrestrial mollusks as intermediate hosts. First-stage larvae (L1) are shed in rodent feces and infect mollusks, where they develop into third-stage larvae (L3), the infective form for definitive hosts. Human infection occurs accidentally, primarily through the ingestion of raw or undercooked mollusks or paratenic hosts, including crustaceans, amphibians, and reptiles, as well as through vegetables contaminated with larval secretions.^4^ In humans, L3 larvae do not complete their life cycle and instead migrate to the central nervous system, where their death triggers an inflammatory response that leads to eosinophilic meningitis (neuroangiostrongyliasis).^5^

Current therapeutic options for nematode infections, such as albendazole, show limited efficacy against migrating larval stages within host tissues.^6–8^ These limitations highlight the need for new compounds with improved efficacy and safety profiles.^9^ In addition to its clinical relevance, *A. cantonensis* has been widely used as an experimental model due to its physiological similarities to other parasitic nematodes and its suitability for standardized *in vitro* assays.^10–13^

Natural products remain a major source of bioactive compounds and have played a central role in drug discovery.^14,15^ In this context, species of the genus *Baccharis* (Asteraceae) are widely recognized in traditional medicine for their anti-inflammatory, antioxidant, and antiparasitic properties.^16^ Extracts and isolated metabolites from species such as *Baccharis lateralis* and *Baccharis retusa* have demonstrated antiparasitic activity, highlighting the potential of this genus as a source of new therapeutic candidates.^17,18^ Notably, *B. lateralis*, a species native to Brazil, is rich in bioactive flavonoids with pharmacological potential.^19^

Sakuranetin is a flavanone widely distributed across plant families, including Asteraceae, and has been reported to exhibit a broad range of biological activities, such as antimicrobial, antifungal, antiparasitic, and antiviral effects.^20,21^ Its antiparasitic activity has been demonstrated against *Entamoeba histolytica*,^22^ *Leishmania* spp. and *Trypanosoma cruzi*.^17^ However, its activity against nematode parasites remains unexplored. To address this gap, this study evaluated the anthelmintic potential of sakuranetin against first-stage (L1) and third-stage (L3) larvae of *A. cantonensis*. Its activity was compared with that of albendazole and pyrantel pamoate, while its safety profile was assessed in mammalian cell lines and in the free-living nematode *Caenorhabditis elegans*. In addition, fluorescence-based viability assessment was used to further characterize larval susceptibility, and *in silico* analyses were conducted to estimate potential toxicological effects.

## 2 Materials and Methods

### 2.1 General Experimental Procedures

NMR spectra were recorded on a Bruker Ascend Evo 600 spectrometer (Bruker, Billerica, MA, USA), respectively, operating at 600 and 150 MHz for ^1^H and ^13^C nuclei, using CD_3_OD (Sigma-Aldrich—St. Louis, MO, USA) as solvent and internal standard. ESI-HRMS spectra were measured on a Bruker Daltonics MicroTOF QII spectrometer. Silica gel 60, 63–210 mesh (Merck—Rahway, NJ, USA) and Sephadex LH-20 (GE Healthcare—Chicago, IL, USA) were used for column chromatography while silica gel F254 (Macherey-Nagel—Düren, NRW, Germany) was used for analytical TLC.

### 2.2 Plant Material

Leaves of *B. lateralis* Baker (Asteraceae) were collected in May 2015 in Campos do Jordão, São Paulo, Brazil (22°42′38.7″S; 45°35′10.1″W). The species was taxonomically identified and a voucher specimen (no. 220669) was deposited at the SPF Herbarium of the *Instituto de Botânica de São Paulo*. The study was registered in the Brazilian National System for the Management of Genetic Heritage and Associated Traditional Knowledge (SisGen; code A4123E4).

### 2.3 Extraction and Isolation of Sakuranetin

Dried leaves (500 g) were defatted with hexane (3 × 2 L) and subsequently extracted with MeOH (6 × 3 L) at room temperature. The crude MeOH extract (24.1 g) was successively partitioned into hexane and CH_2_Cl_2_ to afford, respectively, 0.5 and 10.7 g of each phase. Part of the CH_2_Cl_2_ phase (10.0 g) was subjected to silica gel column chromatography eluted with increasing amounts of EtOAc in hexane to give 79 fractions which were pooled together in five groups (A–E) after TLC analysis. Group B (977 mg) was chromatographed over Sephadex LH-20 using hexane:CH_2_Cl_2_ (1:4), CH_2_Cl_2_:acetone (3:2 and 1:4) and pure acetone as eluents to afford 618 mg of sakuranetin.

#### 2.3.1 Sakuranetin (5,4′-Dihydroxy-7-methoxyflavanone)

^1^H NMR (600 MHz, CD_3_OD) δ_H_: 7.26 (d, *J* = 8.5 Hz, H-2′/H-6′), 6.82 (d, *J* = 8.5 Hz, H-3′/H-5′), 5.97 (s, H-6/H-8), 5.24 (dd, *J* = 13.0 and 3.0 Hz, H-2), 3.74 (s, 7-OMe), 3.05 (dd, *J* = 17.2 and 13.0 Hz, H-3a), 2.66 (dd, *J* = 17.2 and 3.0 Hz, H-3b). ^13^C NMR (150 MHz, CD_3_OD) δ_C_: 197.6 (C-4), 168.9 (C-4′), 164.7 (C-7), 164.0 (C-5), 158.4 (C-8a), 130.3 (C-1′), 127.7 (C-2′/C-6′), 115.8 (C-3′/C-5′), 103.5 (C-4a), 94.5 (C-6), 93.6 (C-8), 79.8 (C-2), 55.7 (7-OMe), 43.3 (C-3). ESI-HRMS *m*/*z* 287.0922 [M + H]^+^ (calculated for C_16_H_15_O_5_, 287.0920).

### 2.4 Parasite and Animal Maintenance

The life cycle of *A. cantonensis* (NPDN-AC strain) was maintained at the Research Center on Neglected Diseases, Guarulhos University, using Wistar rats (*Rattus norvegicus*) as definitive hosts and *Biomphalaria glabrata* and *Achatina fulica* as intermediate hosts under controlled conditions (22 ± 1 °C; 50–60% humidity, ad libitum feeding). Both mollusk species are routinely used for parasite maintenance in our laboratory. However, for the present study, L3 larvae used in the *in vitro* assays were obtained exclusively from experimentally infected *A. fulica*, owing to its greater capacity to sustain high parasite burdens, which facilitates the recovery of the large numbers of L3 larvae required for anthelmintic assays.

### 2.5 Anthelmintic Assays with *A. cantonensis* *Larvae* (L1 and L3)

First-stage (L1) larvae of *A. cantonensis* were obtained from feces of infected rats using the Rugai sedimentation technique^23^ and washed in RPMI 1640 supplemented with antibiotics. Third-stage larvae (L3) were obtained from experimentally infected *A. fulica* by artificial digestion in HCl–pepsin solution,^24^ followed by sedimentation using a modified Rugai method.^23^

Approximately 50 larvae were distributed per well in 96-well plates. Sakuranetin and albendazole were tested starting at 100 μM using serial dilutions to determine EC_50_ values. Plates were incubated at 21 °C, and untreated larvae served as controls. Larval motility was assessed after 24 h and classified into four categories (immobile, intermittent, slow, or highly active). Motility analyses were performed independently by two evaluators using standardized scoring criteria, supported by image acquisition through a microscope-coupled camera system and software-assisted assessment of motility patterns (Motic AE2000, Richmond, BC, Canada). Compounds were considered active when ≥ 60% of larvae were immobile.^10^

### 2.6 Phenotypic and Viability Analyses

Larvae of *A. cantonensis* exposed to sakuranetin, albendazole, or pyrantel pamoate were examined by light microscopy using an inverted microscope (Motic AE2000). Images were captured and analyzed using Motic Images Plus 3.0 software. Larval body length was measured from acquired images, with at least six larvae per group randomly selected in each independent experiment for quantitative characterization of the observed phenotypic changes.^25^ Data were expressed as mean ± standard deviation.

Larval viability was further assessed by fluorescence microscopy using propidium iodide (PI) staining.^26^ After treatment, PI was added directly to each well, followed by incubation for 30 min in the dark. Larvae were analyzed under a fluorescence microscope, and fluorescence was used as an indicator of loss of viability. Albendazole and pyrantel pamoate were used as reference drugs. Larvae exposed to −80 °C served as positive controls, while untreated larvae were used as negative controls. Images were processed using ImageJ software, and mortality was expressed as the percentage of PI-positive larvae.

### 2.7 Cytotoxicity Assay in Mammalian Cell Lines

HaCaT (human keratinocytes) and SH-SY5Y (human neuroblastoma) cell lines were obtained from the Banco de Células do Rio de Janeiro (BCRJ, Duque de Caxias, RJ, Brazil), while Vero cells (monkey kidney epithelial cells; ATCC CCL-81) were purchased from the American Type Culture Collection (Manassas, VA, USA). SH-SY5Y cells were cultured in a 1:1 mixture of Dulbecco’s modified Eagle’s medium (DMEM) and Ham’s F12 medium supplemented with 10% fetal bovine serum (FBS). HaCaT and Vero cells were maintained in DMEM supplemented with 10% FBS. All media were supplemented with penicillin (100 U/mL) and streptomycin (100 μg/mL), and cells were maintained at 37 °C in a humidified atmosphere with 5% CO_2_.^27^

For cytotoxicity assays, cells were seeded into 96-well plates at a density of 2 × 10^3^ cells per well and incubated with sakuranetin (initial concentration 500 μM, followed by serial dilutions). Doxorubicin (15 μM) was used as a positive control, and 0.5% DMSO served as a negative control. Cell viability was evaluated after 24 h using the MTT assay.^28^ Briefly, MTT solution was added and incubated for an additional 4 h, followed by absorbance measurement at 595 nm. Cell viability was expressed as a percentage relative to control wells.^29^

### 2.8 Toxicity Assay in *C. elegans*

*C. elegans* (Bristol N2 strain) was cultured on nematode growth medium (NGM) agar plates seeded with *Escherichia coli* OP50 as a food source.^30^ Synchronized fourth-stage larvae (L4) were transferred to 96-well plates (approximately 80 larvae per well) containing M9 medium.

Sakuranetin was tested at concentrations up to 1000 μM. Ivermectin (10 μM) was used as a positive control, and 0.5% DMSO served as a negative control. After 24 h incubation at 21 °C, larval viability was assessed based on motility. Toxicity was defined as ≥60% of larvae being immobile.^31–33^

### 2.9 *In Silico* Toxicity Prediction

Acute oral toxicity (LD_50_), GHS classification, and organ-specific toxicity end points were predicted using the ProTox-3.0 platform.^34^

### 2.10 Statistical Analysis

EC_50_ and CC_50_ values were calculated using nonlinear regression (GraphPad Prism 8.0). Statistical comparisons were performed using one-way ANOVA followed by Tukey’s post hoc test. A *p*-value <0.05 was considered statistically significant.^35^

### 2.11 Ethical Statement

All animal procedures were approved by the Institutional Animal Care and Use Committee of Guarulhos University (protocol no. 064/24).

## 3 Results

### 3.1 Chemical Characterization of Sakuranetin

The structure of sakuranetin (Figure 1) was confirmed by comparison of obtained NMR and ESI-HRMS data with those previously reported.^17^ The compound purity was estimated at 99% based on HPLC analysis.

**Figure 1:**
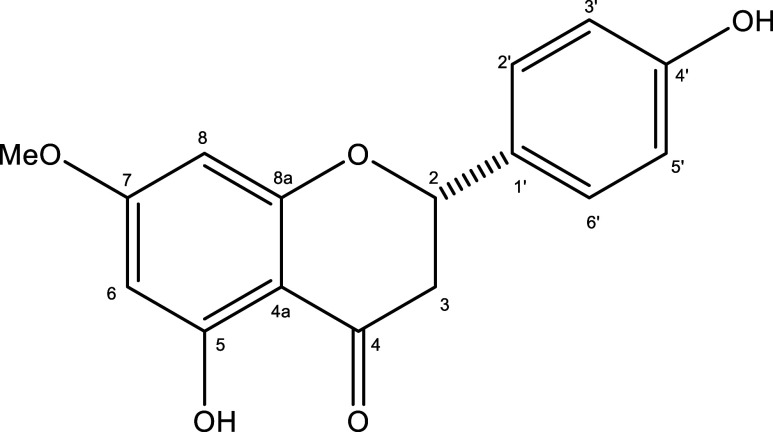
Chemical structure of sakuranetin, a naturally occurring flavanone from *B. lateralis*.

### 3.2 Anthelmintic Activity against *A. cantonensis* Larvae

Sakuranetin exhibited *in vitro* anthelmintic activity against both larval stages of *A. cantonensis*, with a marked difference in potency between stages. The compound was significantly more active against L1 larvae (EC_50_ = 8.1 ± 1.3 μM) than against L3 larvae (EC_50_ = 62.7 ± 8.8 μM) (*P* < 0.0001), indicating a clear stage-dependent effect (Table 1). Dose–response curves for all compounds are presented in Figure 2, illustrating the comparative larvicidal profiles of sakuranetin, albendazole, and pyrantel pamoate across the tested concentration range.

**Figure 2:**
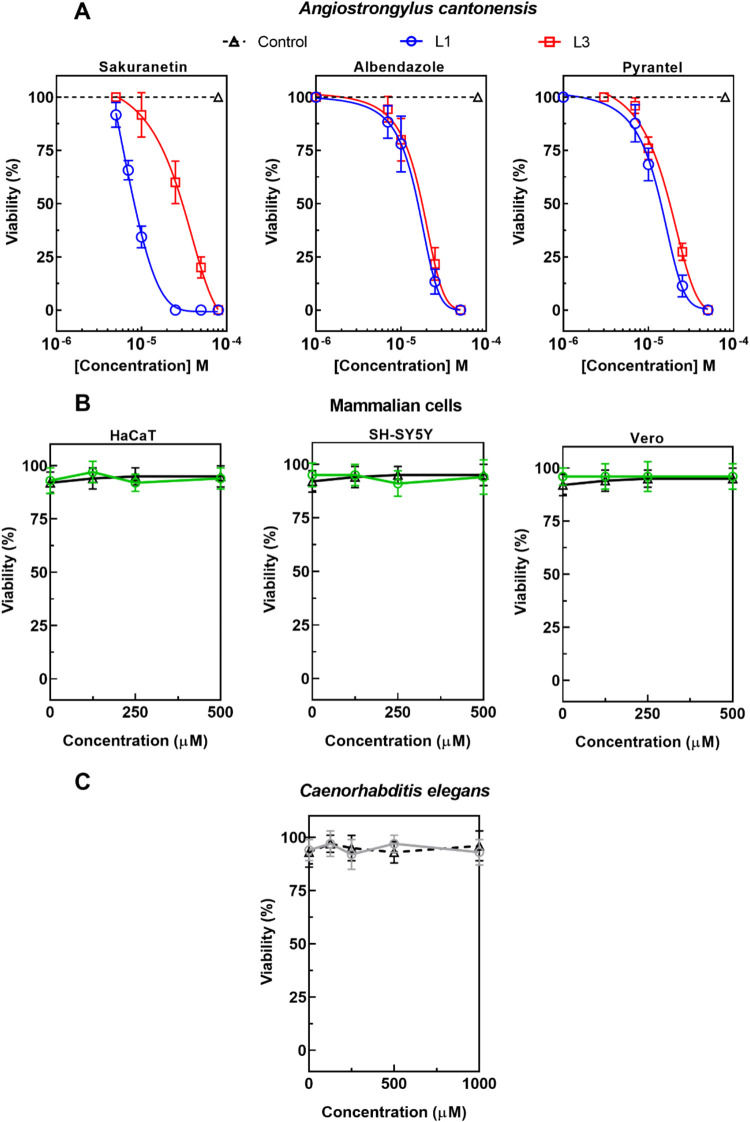
Anthelmintic activity and toxicity profile of sakuranetin and reference drugs. (A) Dose–response curves of sakuranetin, albendazole, and pyrantel pamoate against first-stage (L1) and third-stage (L3) larvae of *A. cantonensis* after 24 h of exposure. (B) Cytotoxicity profiles in mammalian cell lines (HaCaT, SH-SY5Y, and Vero). (C) Toxicity assessment in *C. elegans*. Black triangles represent untreated control groups. Data are presented as mean ± standard deviation (SD) from three independent experiments.

**Table 1:** Anthelmintic Activity and Safety Profile of Sakuranetin and Reference Drugs against *A. cantonensis* ^a^

	*A. cantonensis* (EC_50_, μM)		mammalian cells (CC_50,_ μM)			selectivity index		*C. elegans* (LD_50,_ μM)
Compound	L1	L3	HaCaT	SH-SY5Y	Vero	L1	L3	
Sakuranetin	8.1 ± 1.3*	62.7 ± 8.8**	>500	>500	>500	>60	>7.5	>1000
Albendazole	14.8 ± 1.2	15.3 ± 1.1	>500	>500	>500	>33	>32.5	15.4 ± 1.6
Pyrantel	10.3 ± 0.9	14.4 ± 0.8	>500	>500	>500	>48	>34.5	24.7 ± 2.9

a EC_50_ values were determined for first-stage (L1) and third-stage (L3) larvae. Cytotoxicity (CC_50_) was evaluated in mammalian cell lines (HaCaT, SH-SY5Y, and Vero). The selectivity index was calculated as the ratio between CC_50_ and EC_50_ values. Toxicity in *C. elegans* is expressed as LD_50_ values based on larval viability. Data are presented as mean ± standard deviation (SD) from three independent experiments. *P* < 0.01 compared to albendazole. **P < 0.0001 between L1 and L3 for sakuranetin.

Under the same experimental conditions, albendazole and pyrantel pamoate showed EC_50_ values of approximately 15 μM and 10 μM, respectively, against L1 larvae (Table 1). Sakuranetin was significantly more potent than albendazole at this stage (*P* < 0.01) and displayed comparable activity to pyrantel pamoate. In contrast, albendazole and pyrantel pamoate maintained similar levels of activity against L3 larvae, whereas sakuranetin showed a marked reduction in potency. These findings confirm a pronounced stage-dependent profile for sakuranetin.

### 3.3 Cytotoxicity and Overall Toxicity Profile

Cytotoxicity was assessed in human HaCaT and SH-SY5Y cell lines, together with Vero cells of nonhuman (monkey kidney) origin. No cytotoxic effects of sakuranetin were observed at concentrations up to 500 μM in any of the tested cell lines. Similar profiles were observed for albendazole and pyrantel pamoate. Cytotoxicity data for all compounds across the tested concentration range are presented in Figure 2. Based on these data, sakuranetin exhibited high selectivity indices (SI > 60 for L1), exceeding those of albendazole (SI > 33) and pyrantel pamoate (SI > 48) (Table 1).

In the *C. elegans* model, sakuranetin showed no detectable toxicity up to 1000 μM (LD_50_ > 1000 μM). In contrast, albendazole and pyrantel pamoate displayed measurable toxicity, with LD_50_ values of 15.4 μM and 24.7 μM, respectively. The toxicity profiles in *C. elegans* are also shown in Figure 2.

To complement the experimental data, *in silico* toxicity predictions were performed using the ProTox-3.0 platform (Figure 3). Sakuranetin showed a predicted oral LD_50_ of 2000 mg/kg in rats, higher than albendazole (970 mg/kg) and pyrantel pamoate (1190 mg/kg), suggesting lower acute systemic toxicity. No alerts for hepatotoxicity or neurotoxicity were identified for sakuranetin, whereas albendazole and pyrantel pamoate showed predicted hepatotoxicity, and albendazole additionally showed potential neurotoxicity. Together, these findings indicate low toxicity across the experimental models evaluated, supported by favorable *in silico* toxicity predictions for sakuranetin.

**Figure 3:**
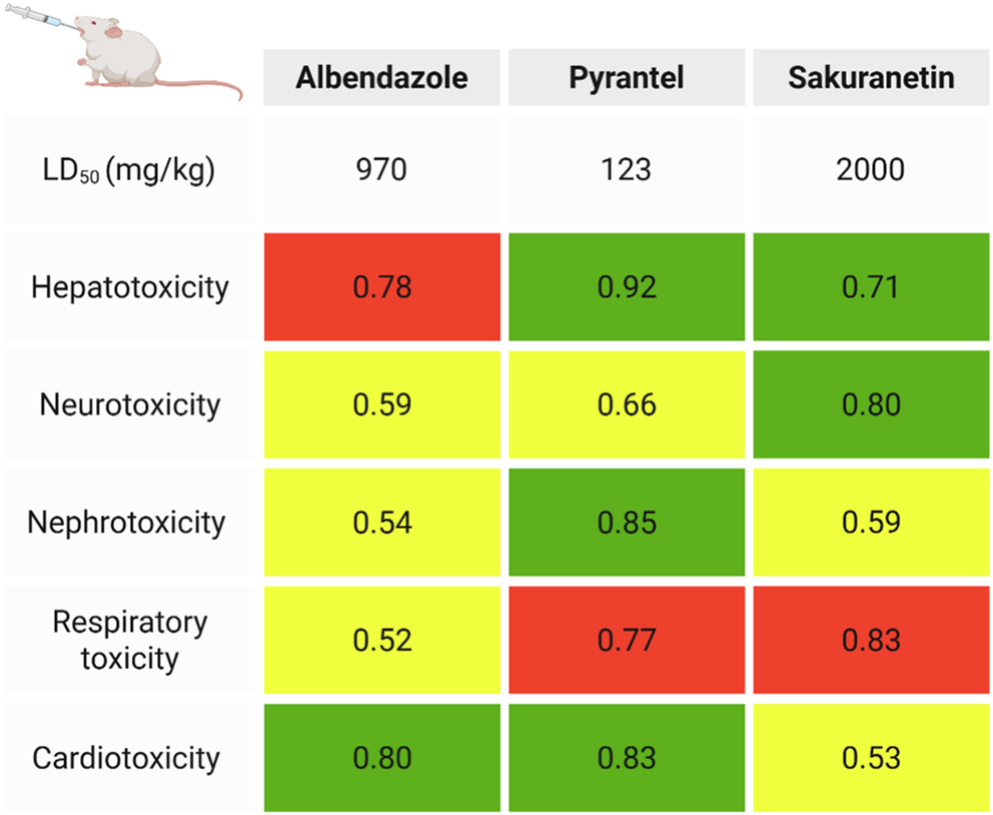
*In silico* toxicity prediction of sakuranetin and reference drugs. (Top row) Predicted acute oral toxicity (LD_50_ mg/kg) in rats, indicating lower systemic toxicity for sakuranetin compared to albendazole and pyrantel pamoate. (Bottom rows) Heatmap of predicted organ-specific toxicity end points, including hepatotoxicity, neurotoxicity, nephrotoxicity, respiratory toxicity, and cardiotoxicity. Color scale: green (inactive), yellow (moderate toxicity, <0.70), and red (high toxicity, ≥0.70).

### 3.4 Phenotypic and Fluorescence-Based Viability Alterations in *A. cantonensis* Larvae

Exposure to sakuranetin induced pronounced phenotypic changes in *A. cantonensis* larvae, which differed from those observed with albendazole (Figure 4). Larvae treated with albendazole largely retained an elongated morphology, whereas those exposed to sakuranetin and pyrantel pamoate exhibited marked body contraction and coiling. Morphometric analysis confirmed these observations.

**Figure 4:**
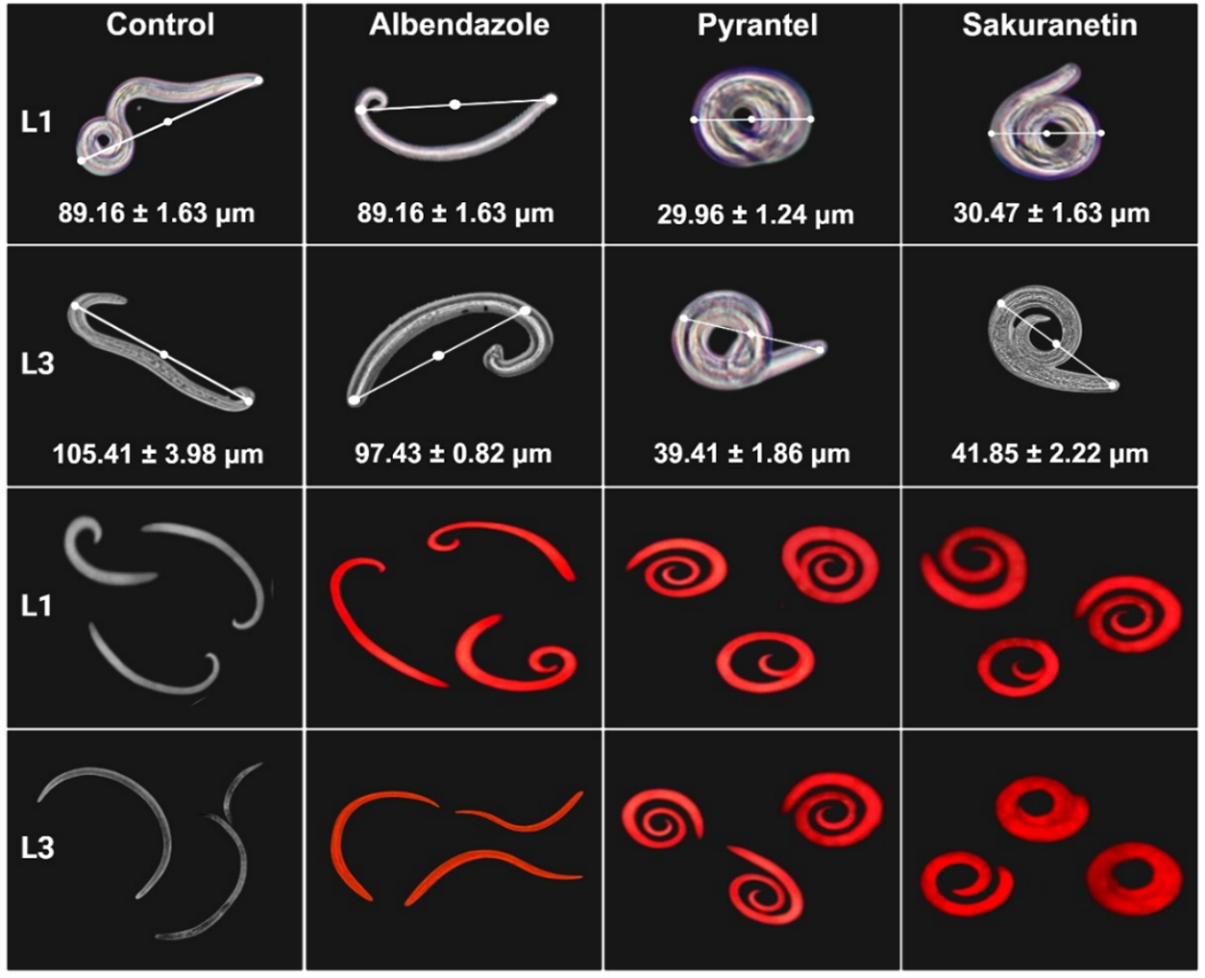
Phenotypic and fluorescence-based viability alterations in *A. cantonensis* larvae following treatment. Upper panels: Representative light microscopy images of first-stage (L1) and third-stage (L3) larvae in control and treated groups (albendazole, pyrantel pamoate, and sakuranetin), illustrating distinct morphological profiles. Albendazole-treated larvae retained an elongated morphology, whereas sakuranetin- and pyrantel pamoate-treated larvae exhibited marked body contraction and coiling. Corresponding morphometric analysis (mean ± SD, μm) shows a modest reduction in larval length with albendazole and a pronounced reduction with sakuranetin and pyrantel pamoate. Lower panels: Fluorescence microscopy images of L1 and L3 larvae, respectively, after propidium iodide staining, illustrating treatment-associated staining patterns used for viability assessment.

In control groups, mean larval length was 98.78 μm (L1) and 105.41 μm (L3). Albendazole treatment resulted in a modest but significant reduction in length (89.16 ± 1.63 μm for L1 and 97.43 ± 0.82 μm for L3; *P* < 0.05). In contrast, sakuranetin and pyrantel pamoate induced a pronounced reduction in larval length, reaching 30.47 and 29.96 μm (L1), and 41.85 and 39.41 μm (L3), respectively (*P* < 0.0001).

Fluorescence microscopy revealed treatment-dependent differences in propidium iodide (PI) staining patterns between larval stages (Figures 4 and 5). Quantitative analysis demonstrated that sakuranetin induced significantly higher fluorescence intensity in L1 larvae compared to L3 (*P* < 0.001), consistent with their increased susceptibility. In contrast, albendazole showed an opposite pattern, with higher fluorescence intensity in L3 than in L1. No significant differences between larval stages were observed for pyrantel pamoate. These findings indicate that fluorescence responses vary according to both the compound and larval stage.

**Figure 5:**
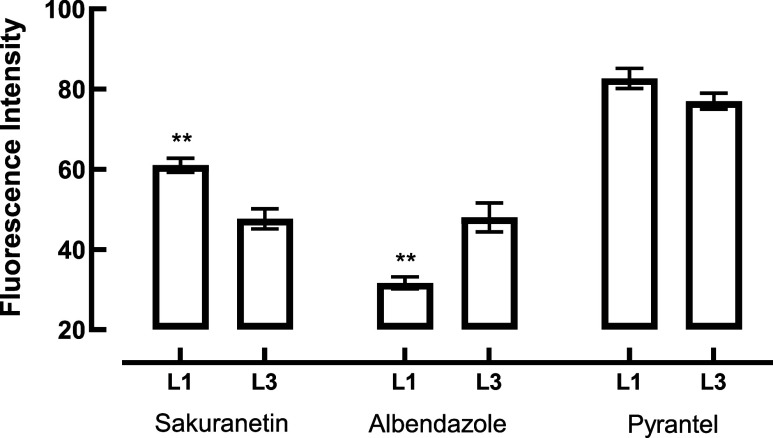
Quantitative analysis of fluorescence intensity in *A. cantonensis* larvae following treatment. Fluorescence intensity was measured in first-stage (L1) and third-stage (L3) larvae after exposure to sakuranetin, albendazole, and pyrantel pamoate, based on propidium iodide staining. Increased fluorescence intensity reflects reduced larval viability. Data were obtained from fluorescence images and quantified using ImageJ software. Bars represent mean ± standard deviation (SD) from three independent experiments. *P* < 0.001 indicates significant differences between L1 and L3 within the same treatment; no significant differences were observed for pyrantel pamoate.

## 4 Discussion

The growing burden of zoonotic helminth infections, combined with the limitations of currently available anthelmintics, underscores the need for new therapeutic agents.^2,9^ In this context, the present study identifies sakuranetin as a promising natural product with anthelmintic activity against *A. cantonensis*.

A key finding of this study is the marked stage-dependent activity of sakuranetin. The pronounced difference in susceptibility between L1 and L3 larvae highlights developmental stage as an important determinant of drug responsiveness in *A. cantonensis*. This pattern is particularly relevant, as early larval stages are critical for parasite establishment and migration within the host.^3,4^ Similar stage-dependent responses have been reported for other anthelmintics, reflecting physiological and structural differences between larval stages that may influence drug uptake or target accessibility, including variation in cuticle composition and metabolic activity.^36,37^ The fluorescence-based viability assays further support the existence of stage-dependent differences in larval susceptibility.

The potency of sakuranetin against L1 larvae (EC_50_ = 8.1 μM) is comparable to that reported for other natural products evaluated in *A. cantonensis*. Compounds such as piplartine (EC_50_ = 8.3 μM),^31^ spathulenol (EC_50_ = 7.6 μM),^25^ and cubebin (EC_50_ = 4.7 μM)^33^ have demonstrated similar or higher activity against L1 larvae, while 1,10-phenanthroline-5,6-dione derivatives have shown activity within a broader range (EC_50_ = 6.4–25.2 μM).^12^ Notably, differences in stage-dependent activity have also been reported for some of these compounds, reinforcing the importance of considering larval stage when evaluating anthelmintic efficacy. Although direct comparisons should be interpreted with caution due to differences in experimental conditions, these data place sakuranetin among the most active compounds reported against early larval stages of *A. cantonensis*.

The activity profile of sakuranetin relative to established anthelmintics further supports its relevance as a natural product scaffold for nematode drug discovery. However, its reduced activity against L3 suggests that its efficacy may be limited against infective larval stage relevant to human disease.^6^ This observation represents an important translational consideration and underscores the need to account for stage-specific susceptibility when evaluating and prioritizing new anthelmintic candidates.

One of the most notable findings of this study is the favorable selectivity profile of sakuranetin. The compound showed no detectable cytotoxicity in mammalian cell lines and no toxicity in the free-living nematode *C. elegans* at the highest tested concentrations. In contrast, albendazole and pyrantel pamoate, although noncytotoxic in mammalian cells, exhibited measurable toxicity in *C. elegans*. This differential profile suggests that sakuranetin may exhibit greater activity against parasitic larvae than against the experimental nonparasitic models evaluated, representing a potentially desirable feature for candidate anthelmintics.

The phenotypic alterations induced by sakuranetin provide additional insight into its biological effects. The pronounced contraction and coiling observed in treated larvae closely resemble the phenotype induced by pyrantel pamoate, a known nicotinic receptor agonist that causes spastic paralysis.^38^ Although the mechanism of action of sakuranetin was not directly investigated in this study, these similarities raise the hypothesis that it may interfere with neuromuscular function or related physiological pathways.^39^ In addition, previous studies have shown that sakuranetin can modulate neurotransmitter systems and cellular stress responses, including oxidative stress and inflammatory pathways,^40^ suggesting a broader biological profile that may contribute to the observed larval phenotypes. Future studies aimed at elucidating its mechanism of action should include approaches to assess possible effects on neuromuscular signaling, mitochondrial function, oxidative stress, and larval drug uptake or permeability, particularly to clarify the marked differences in susceptibility observed between L1 and L3 larvae.

The *in silico* toxicity predictions were consistent with the experimental findings, supporting a comparatively favorable predictive toxicological profile for sakuranetin. Although sakuranetin showed a higher predicted LD_50_ than the reference drugs and no alerts for hepatotoxicity or neurotoxicity, these predictions should be interpreted cautiously, as computational models may not fully capture the complexity of biological systems.^14^

Although these findings are promising, some limitations should be acknowledged. The study was restricted to *in vitro* assays, and the efficacy of sakuranetin *in vivo* remains to be determined. In addition, the substantially lower activity observed against L3 larvae, the infective stage associated with human neuroangiostrongyliasis, highlights an important translational limitation and suggests that further optimization may be required, including through structural modification, formulation approaches, or combination strategies.

In conclusion, sakuranetin emerges as a promising anthelmintic candidate with stage-dependent activity, low toxicity in the experimental models evaluated, and favorable predictive toxicological properties. Its activity against parasitic larvae, combined with low toxicity in mammalian cells and in a free-living nematode model, supports further investigation toward the development of new therapeutic strategies. Furthermore, while the present results were obtained using *A. cantonensis*, this parasite has been widely employed as a model for anthelmintic drug discovery, supporting the broader relevance of these findings for parasitic nematodes.
